# You sure about that? The effects of textual and image-based Skepticism on belief in dubious social-media claims

**DOI:** 10.1186/s41235-026-00725-x

**Published:** 2026-03-30

**Authors:** Emily R. Spearing, Juliana Garib Jankauskas, Eryn J. Newman, Ullrich K. H. Ecker

**Affiliations:** 1 School of Psychological Science, University of Western Australia (M304), Perth, Australia; 2 School of Medicine and Psychology, Australian National University, Canberra, Australia; 3 Public Policy Institute, University of Western Australia, Perth, Australia

**Keywords:** Skepticism, Social media, Fact-checking, Misinformation

## Abstract

On social media, people often react to posts using both written comments and images. While prior work has shown that these reactions can influence belief in claims, it has often conflated signals of (dis)endorsement with justifications of dissent. It is therefore unclear whether skeptical comments that express doubt about a claim’s veracity without providing informational content can reduce claim belief. We tested whether pure text- or image-based expressions of skepticism reduce belief in dubious claims, and how skepticism compares with non-evidence-supported rebuttals that explicitly declare a claim to be false, in the presence versus absence of supportive comments that express general acceptance or agreement with a claim. Participants (*N* = 200) rated their belief in true and false claims presented as social-media posts with varying combinations of supportive, skeptical (textual vs. image-based), or non-evidence-supported negational comments. Results showed that supportive comments were associated with greater claim belief, while skeptical comments reduced belief, with no difference between text and image formats. Direct negations had the strongest corrective effect despite not providing any evidence. Implications are discussed, including the risk of skepticism reducing belief in true claims, underscoring the need for oversight of community-based fact-checking tools on social media.

**Supplementary Information:**

The online version contains supplementary material available at 10.1186/s41235-026-00725-x.

## Introduction

The rise of social media has transformed the contemporary information environment, enabling information—including misinformation^1^—to be accessed and spread at unprecedented speed and scale (Surjatmodjo et al., 2024; Vosoughi et al., 2018). Unlike traditional media, social media allow users to publish content instantly and without verification or editorial oversight. In addition, recent policy changes have seen major platforms replace formal accuracy checks such as professional fact-checking with a reliance on community annotation such as user comments (Augenstein et al., 2025; Borenstein et al., 2025). Public peer reactions may thus play an increasingly influential role in shaping users’ beliefs in online claims, including dubious or false claims. This study investigated how such reactions, delivered in the form of user comments—specifically expressions of skepticism (i.e., expressions of doubt about a claim’s veracity) conveyed through either text or images, or non-evidence-supported negational text comments that explicitly declare a claim to be false—can influence belief in dubious claims; it also examined how the presence of supportive comments expressing general agreement or acceptance of claims affects this process, exploring how social cues combine to shape beliefs.

To understand how online comments might impact beliefs, it is important to consider the influence of social cues on information appraisal. Social cues may lead to bandwagon effects, meaning that truth evaluations may be influenced by a perceived consensus based on informational or normative influence (Cialdini & Goldstein, 2004; Kaplan & Miller, 1987). Previous psychological research has identified the impacts of engagement metrics (e.g., number of views or shares) and peer endorsement (e.g., number of likes) on social media: Exposure to high engagement numbers can increase users’ likelihood of sharing or endorsing content and make them less likely to fact-check it (Avram et al., 2020). Strong endorsement (i.e., a large number of likes) can increase belief in false claims, even following corrective messages; likewise, corrections themselves are more effective when they have received strong endorsement (Butler et al., 2023; but also see Traberg et al., 2024). These findings suggest that numerical social cues can shape both initial belief formation and subsequent belief updating. Moreover, while most social-media platforms do not provide a direct way to express disapproval, such as a “dislike” button, recent work shows that (dis)endorsement (e.g., a large number of dislikes relative to the number of likes) can also decrease belief in claims (Butler et al., 2024; Fay et al., 2025).

In contrast to numerical indicators, textual comments provide a more explicit and nuanced way to express both agreement and dissent, making them a potentially stronger signal of social (dis)endorsement. Supportive comments can increase perceptions of credibility and accuracy, whereas dissenting comments can reduce both belief in information and participants’ willingness to share it, driven at least in part by perceptions of social consensus or lack thereof (Kluck et al., 2019; Lewandowsky et al., 2019; Traberg et al., 2024). Moreover, corrective information in user comments can reduce the influence of misleading social-media posts on health-related beliefs (Bode & Vraga, 2018; Bode et al., 2020) and make users less likely to reproduce misinformation, while making them more likely to produce accurate information (Mason & Rapp, 2026). It is important to note that studies have mainly explored the effect of consistent comments, and few have examined the impact of mixed comment sections containing both supportive and dissenting views, which may more closely resemble real-world social media. However, initial evidence suggests that exposure to multiple, consistently supportive, or dissenting reposts (i.e., articles being reposted with a comment) may lead to corresponding belief change regarding the gist of the reposted article, whereas exposure to a mix of dissenting and supportive reposts may have little impact on belief (Alister et al., 2025). These findings are in line with findings that classic conformity effects are reduced by even a single dissenting voice (Allen & Levine, 1969).

While prior work has yielded important insights into how social endorsement influences belief in claims, its text-oriented nature means that the impact of image-based comments has hitherto been overlooked. Some research has examined the impact of images that accompany a claim on measures of claim belief (e.g., Fenn et al., 2019; Newman & Zhang, 2020; Whitehead et al., 2025), but it is not yet clear how beliefs are shaped by nonverbal comments used to convey users’ (dis)agreement with claims, or whether such pictorial cues are persuasive in the absence of text. Unlike probative images that provide valid evidence about the accuracy of claims, images expressing skepticism (e.g., through facial expressions) are non-probative and could therefore be used to reduce belief in true claims as well as false ones (Newman & Schwarz, 2024). Such images may attract more attention and generate greater engagement than text (Bakhshi et al., 2014; Keib et al., 2018), which may make them more impactful than text-based expressions of doubt. However, images can also be inherently ambiguous. Interpretation of facial expressions, for instance, is shaped by characteristics of expresser, observer, and context (Barrett et al., 2011; Besel & Yuille, 2010; Fang & Li, 2024; Steward et al., 2025), which raises questions about how clearly nonverbal facial cues can convey judgments or impressions of content.

Generally, expressions of skepticism may impact beliefs by promoting reflective information processing, potentially reducing susceptibility to misleading content (Mayo, 2019). This idea is supported by research showing that suspicion regarding the motives behind the dissemination of false claims can reduce claim belief (Lewandowsky et al., 2005) and that prompting more deliberate evaluation or a focus on veracity can reduce belief in misinformation and sharing intentions (Bago et al., 2020; Guess et al., 2020; Pennycook et al., 2021). Moreover, Jalbert and colleagues (2025) found that verbal user comments questioning the truth of information shared on social media (e.g., “How do you know that?” or “Where did you learn this?”) reduced both belief in the information and the likelihood of sharing it, compared to positive or neutral comments. The authors argued that such comments may shift attention toward information veracity, but may also have a social dimension, affecting belief expressions and sharing intentions in accordance with a social norm (also see Andı & Akesson, 2020; Prike et al., 2024).

### The present study

Given the limited research on how skeptical comments function and the underexplored role of image-based cues in this context, the present study aimed to examine how both textual and image-based user expressions of skepticism influence belief in dubious claims. We use the term “dubious claims” to refer to statements that are at least somewhat plausible without having clear evidentiary support, and for which people are unlikely to have strong prior knowledge or preexisting beliefs. Specifically, we investigated whether skeptical comments reduce belief in dubious claims, and whether their impact is influenced by the presence of social endorsement conveyed through supportive textual comments (e.g., “makes sense”). Participants viewed dubious claims (16 true, 32 false) presented as social-media posts and rated their belief in the claims’ accuracy. Claims had a comments section that contained either no dissenting comment, a textual or image-based skeptical comment, or a textual negational comment. Half the claims were also accompanied by three supportive textual comments to signal some consensus in favor of the claim.

Comments were designed to solely indicate support or dissent, without informational or source-related elaboration. This was done because prior research has often used comments that confound social (dis)endorsement with factual arguments or statements that convey emotion or concern the information source. For example, a claim such as “taking vitamin C supplements prevents the flu” might be followed by a dissenting comment that contains additional information such as relevant facts (e.g., “Vitamin C can’t block the flu virus, research shows it has minimal impact on symptoms”) or a statement regarding the credibility of the information source (e.g., “I’m so sick of these fake health influencers spreading lies”). Negational text comments were included to provide a benchmark against which to assess the impact of skepticism. While skepticism merely conveys doubt about a claim’s veracity, negations explicitly declare a claim to be false, thus expressing greater confidence or certainty. A large body of research on fact-checking has demonstrated that providing corrective information reliably reduces belief in false claims, even in a brief format (Clayton et al., 2020; Ecker et al., 2020; Fazio et al., 2024; Kozyreva et al., 2024; Lithander et al., 2021; Prike & Ecker, 2023). To maintain some comparability with skeptical comments, negational comments simply asserted falsity without providing detailed evidence.^2^

The experiment tested a series of hypotheses regarding how different forms of dissent and the presence of supportive comments influence belief in dubious claims. In line with previous research (Alister et al., 2025; Lewandowsky et al., 2019), we predicted that claim belief would be greater in the presence versus absence of supportive comments (H1). Based on prior research suggesting that skeptical comments can reduce belief in questionable content (Jalbert et al., 2025; Kluck et al., 2019), we hypothesized that skeptical comments would lower claim belief relative to control (H2). Given evidence that images draw more attention and engagement than text (Bakhshi et al., 2014; Keib et al., 2018), we expected that image-based skepticism would lead to a greater reduction in belief than textual skepticism (H3); however, this was a tentative hypothesis given the potential ambiguity associated with facial expressions of skepticism. Additionally, we hypothesized that negational comments would produce the largest reduction in belief relative to control, aligning with prior evidence that direct challenges are especially effective at reducing belief in false claims (Ecker et al., 2020; H4). Finally, we expected that the effect of skeptical comments would be stronger when no supportive comments were present (H5): In the absence of social support, a stand-alone skeptical remark may be especially impactful.

## Method

This study used a fully crossed 2 (social endorsement) × 4 (dissent) within-subjects design. Social endorsement was manipulated by presenting claims with or without three supportive text comments. Claims were presented with (1) no dissenting comment (control) or with a dissenting comment that was either (2) a skeptical image, (3) skeptical text, or (4) negational text. This research was approved by the University of Western Australia’s Human Research Ethics Office; materials and data are available at: https://osf.io/5nbcu/.

### Participants

An a priori power analysis conducted using G*Power 3 (Faul et al., 2007) indicated a minimum sample size of 164 participants to detect an effect size of *f* =.15 (*α* =.05 and 1 – *β* =.80), consistent with Butler et al. (2023).^3^ We collected data from 200 participants (118 female, 82 male; *M*_age_ = 45.94 years, SD = 13.20, range = 20–79) from the UK via Prolific (https://www.prolific.co), who had a Prolific approval rating of at least 98%. Self-reported English proficiency ranged from good to excellent; no participants reported fair or poor proficiency.

### Materials

#### Social-media posts

**Fig. 1:**
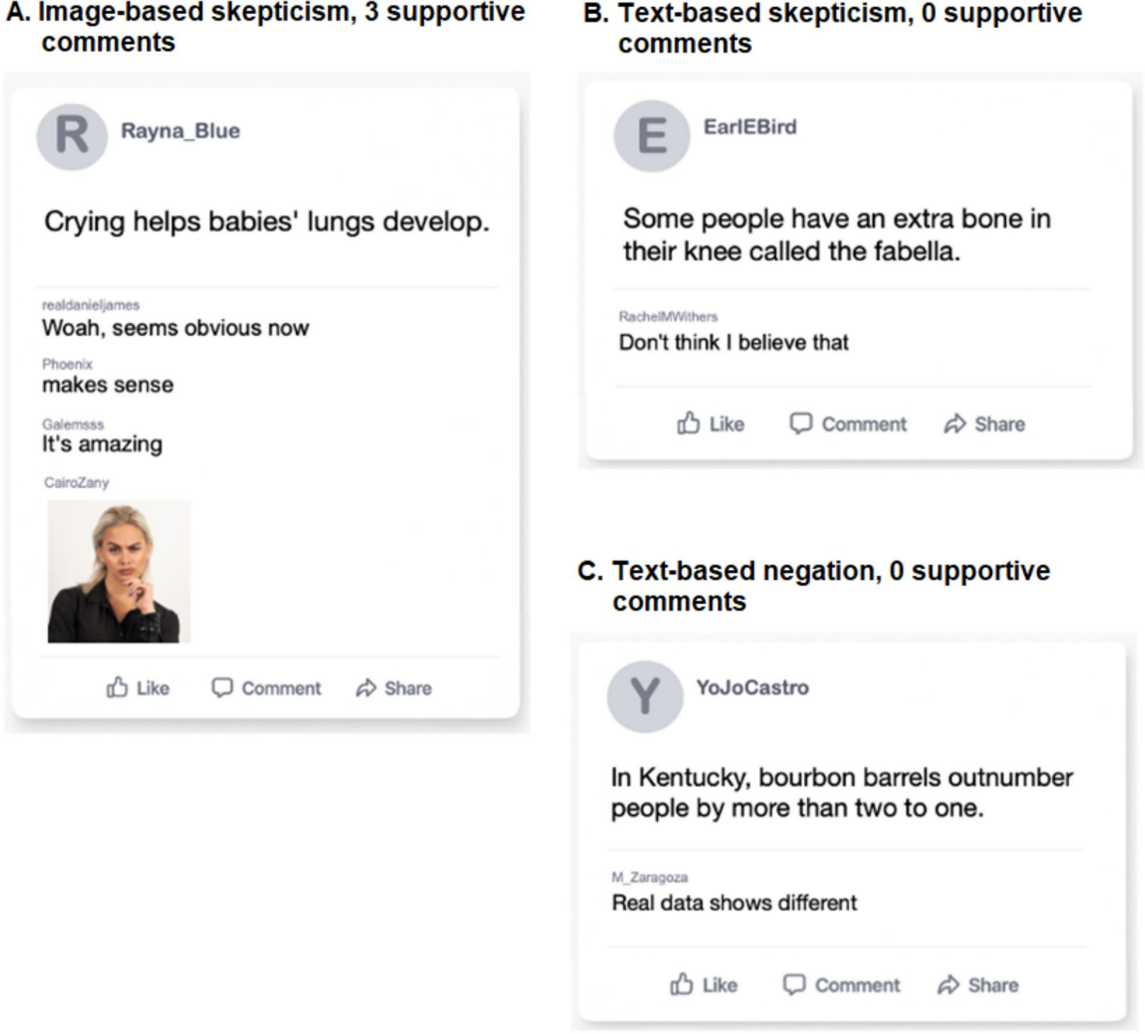
Examples of Social-Media Posts with Comments (Skeptical face image by Adobe Stock contributor rastockinc, available from https://stock.adobe.com/au/images/Portrait-of-serious-woman-in-black-shirt-posing-on-white-background/515888557, used here under standard licence.)

Participants were presented with 48 claims (e.g., “Most people only use between 10 and 50% of their brain capacity”) formatted to resemble social-media posts. The set consisted of 16 true claims and 32 false claims, drawn from previous studies that had established believability ratings for each item (Butler et al., 2023; Prike et al., 2024; Swire-Thompson et al., 2022). We selected claims with mean believability scores between 4.00 and 6.15 on a 0–10 scale (*M* = 4.90, SD = 0.65) to ensure they were moderately plausible while minimizing the likelihood that participants held substantial prior knowledge about their accuracy. Politically polarizing topics were avoided to reduce the influence of preexisting attitudes (Walter & Tukachinsky, 2020). Each social-media post was attributed to a fictitious source, with user names (e.g., “meltaylor84”; “beehive64542”) selected from a previous study (Prike et al., 2024). Example posts are shown in Fig. 1.

The 48 claims were divided into eight sets of six items (each containing two true claims and four false claims) comparable in median believability. To counterbalance claim-condition pairings, the sets were then assigned to experimental conditions using a Latin square design, resulting in eight survey versions. Each participant viewed all 48 claims (six per experimental condition) presented in a randomized order.

#### Text comments

For each claim, we created five text-based comments: three supportive, one skeptical, and one negational. This approach was intended to enhance the ecological validity of the experiment by presenting context-consistent remarks. All text-based comments were brief (one phrase or sentence) to simulate social-media interaction and were devoid of informational content that could justify the (dis)endorsement. Supportive comments expressed agreement or acceptance of the claim (e.g., “Seems right”), skeptical comments conveyed doubt or disbelief (e.g., “That makes no sense at all…”), and negational comments explicitly stated that a claim was false, though this was not accompanied by supporting evidence (e.g., “There’s no record of this.”). Skeptical comments were designed to signal doubt and uncertainty, whereas negational comments were designed to communicate direct rejection of the claim.

When supportive comments were present, the dissenting comment (skeptical or negational) was always displayed last to avoid ambiguity, as presenting supportive comments beneath a dissenting remark could imply agreement with the dissent rather than with the original claim. For example, if a skeptical comment stated “I don’t believe this to be true,” and was followed by a supportive comment such as “I agree with that,” it would be unclear whether the agreement referred to the claim itself or to the skeptical comment.

#### Image comments

To create the skeptical-image comments, we selected a set of 48 images from online stock image databases and open internet sources that appeared to convey skepticism. This set included 32 photographs of human facial expressions and 16 cartoon-style illustrations or emojis. We then conducted a pilot study with a separate sample of *N* = 50 UK-based participants recruited via Prolific, who rated the extent to which each image conveyed skepticism on a scale from 0 (*not skeptical at all*) to 10 (*extremely skeptical*). The twelve highest-rated images (mean scores ranging from *M* = 6.89 to *M* = 7.46) were then selected for use in the main experiment and divided into two sets of six, each containing five face photos and one cartoon image. Half of the eight claim sets were paired with images from the first set, and the other half with images from the second set. Specific images were assigned to claims such that across survey versions, each skeptical image was paired with four claims of comparable mean believability. Each participant viewed all 12 selected skeptical images but saw each image only once.

### Procedure

Participants viewed an ethics-approved information sheet and provided informed consent, then provided some demographic information (i.e., age, gender, English proficiency). They were then randomly assigned to one of the eight survey versions and rated the perceived accuracy of each claim on an 11-point scale ranging from 0 (*very likely inaccurate*) to 10 (*very likely accurate*) in a random order, with each post presented on a separate page. Finally, participants were fully debriefed following best-practice guidelines (Greene et al., 2022) and given access to a full list of the claims, which indicated whether each claim was true or false. Participants received £1.65 for completing the experiment, which took approximately 10 min.

## Results

**Fig. 2:**
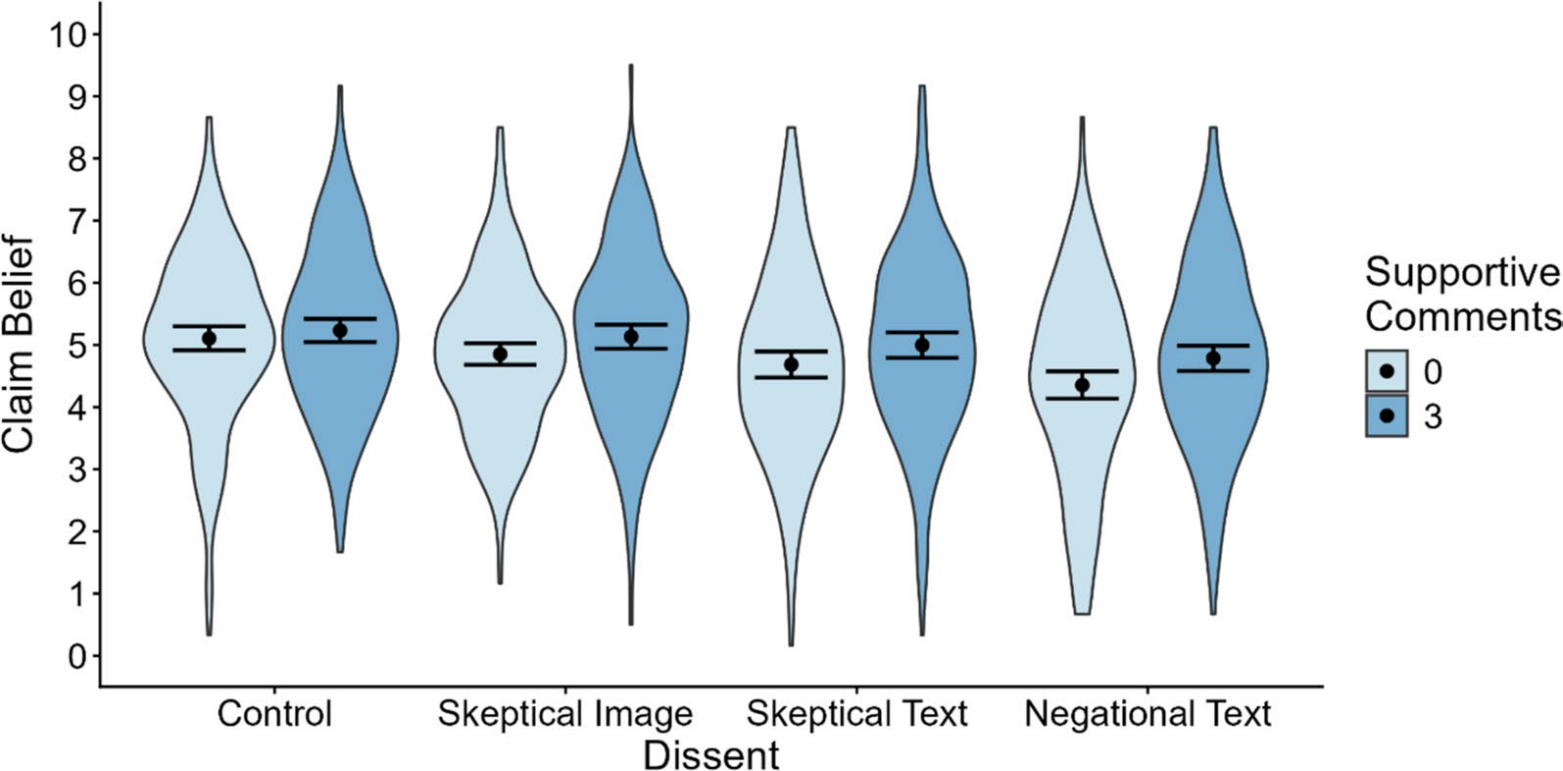
Mean Claim Belief by Dissent Condition. Error bars denote the 95% CI around the mean. Means are collapsed across true and false claims

To examine the impact of dissenting comments on belief in dubious claims, we ran a 2 (social endorsement: 0 supportive comments, 3 supportive comments) × 4 (dissent: control, skeptical image, skeptical text, negational text) mixed-effects model on belief ratings (i.e., perceived claim accuracy) across both true and false claims,^4^ with random intercepts for each participant and claim.^5^ The model was fitted using the *afex* package v1.4–1 in R 4.4.1, and planned contrasts were conducted using the *emmeans* package v1.10.4. To quantify evidence for the observed effects, Bayesian independent *t*-tests were conducted on participant means using the *BayesFactor* package v0.9.12–4.7. Mean claim belief ratings for each condition are shown in Fig. 2

The model revealed a significant effect of social endorsement, *F*(1, 9346) = 28.16, *p* <.001, *d* = 0.11. Participants expressed greater belief in claims accompanied by (three) supportive comments than claims presented without supportive comments, so H1 was supported. Exploratory post hoc contrasts indicated, however, that this effect was nonsignificant in the control condition, *F*(1, 9346) = 1.26, *p* =.263, *d* = 0.05 (a Bayesian *t-test* revealed moderate support for the null hypothesis; *BF*_01_ = 3.86), such that supportive comments only had a significant impact on belief in the presence of dissent (for the experimental conditions: all *F*(1, 9346) ≥ 6.63, *p*s ≤.010, *d* ≥ 0.11, *BF*_10_ ≥ 4.27). There was also a significant effect of dissent, *F*(3, 9346) = 21.64, *p* <.001, *d* = 0.17. Consistent with H2, dissenting comments significantly reduced belief in dubious claims relative to control, whether they included a skeptical image, *F*(1, 9346) = 5.27, *p* = *.*022, *d* = 0.07 (although a Bayesian *t*-test yielded only anecdotal evidence; *BF*_10_ = 1.87), skeptical text, *F*(1, 9346) = 17.54, *p* < *.*001, *d* = 0.12 (*BF*_10_ = 111.04), or negational text, *F*(1, 9346) = 60.50, *p* <.001, *d* = 0.23 (*BF*_10_ > 1000). The interaction between social endorsement and dissent was nonsignificant, *F*(3, 9346) = 1.42, *p* =.236, *d* = 0.04.

To examine which types of dissenting comments were most effective at reducing belief in dubious claims, we conducted planned contrasts comparing the reduction in claim belief (relative to control) across dissent conditions. The reduction in claim belief did not significantly differ between the skeptical-image and skeptical-text conditions, *F*(1, 9346) = 3.58, *p* =.058, *d* = 0.06 (BF_01_ = 34.94), so H3 was not supported. However, negational text comments produced a larger reduction in claim belief than both skeptical images, *F*(1, 9346) = 30.06, *p* <.001, *d* = 0.16 (BF_10_ > 1000), and skeptical text, *F*(1, 9346) = 12.89, *p* <.001, *d* = 0.10 (BF_*10*_ = 66.30), supporting H4.

Finally, to directly examine whether the impact of dissenting comments depended on the presence of supportive comments, we conducted planned contrasts comparing the belief-reducing impact of dissenting comments (vs. control) across the two levels of social endorsement. For both skeptical images, *F*(1, 9364) = 1.06, *p* =.304, *d* = 0.06 (BF_01_ = 4.43), and skeptical text, *F*(1, 9364) = 1.61, *p* =.204, *d* = 0.07 (BF_01_ = 3.79), the result was nonsignificant, suggesting a similar reduction in claim belief when expressions of skepticism were accompanied by supportive comments compared to when they were not. Thus, H5 was not supported.

## Discussion

The present study tested whether textual and image-based expressions of skepticism can reduce belief in dubious claims presented in a social-media format. It also assessed how skeptical comments compare with direct negations, and whether effects are influenced by the presence of supportive comments. Results showed that skeptical comments reduced belief (relative to a control condition), regardless of their textual versus image-based format, although their effect was generally smaller than the effect of negational comments. Supportive comments were generally associated with higher belief ratings—even though they were rather generic (e.g., “Amazing!” “Makes sense”). While the size of these effects was small, their implications should not be underestimated—in online environments, where large numbers of users are exposed to claims and comments, even modest effects can have a meaningful impact at scale.

The fact that brief skeptical comments reduced belief in dubious claims suggests that singular dissent signals can influence claim perceptions, even when they are devoid of informational content. This finding builds on previous work which has mainly demonstrated effects of dissent when it was accompanied by some elaboration or justification (e.g., Alister et al., 2025; Lewandowsky et al., 2019), or when it was expressed numerically (i.e., through large numbers of “dislikes”; Butler et al., 2024; Fay et al., 2025); it is also consistent with Jalbert et al. (2025), who found that user comments questioning the veracity of a social-media claim reduced both belief and willingness to share.

Expressions of skepticism may impact beliefs by acting as prompts for deliberation; this idea is supported by previous research showing that accuracy prompts can encourage individuals to reflect more critically on claims, thereby reducing misinformation susceptibility (e.g., Bago et al., 2020; Pennycook et al., 2021). Even minimalist expressions of doubt may serve as reminders to question the plausibility of information, prompting more analytical processing. When presented alongside supportive comments, expressions of skepticism may also interrupt heuristic processing based on perceived social consensus—even a single skeptical comment may be sufficient to undermine the perception of unanimity (Allen & Levine, 1969), prompting a shift to more analytical processing to evaluate a claim on its own merit.

To the best of our knowledge, this is the first study to examine the effect of user comments expressed through images on claim belief. Both textual and image-based skeptical comments reduced belief; although there was no significant difference between formats, we note that the effect size for image-based skepticism was approximately half that of textual skepticism, suggesting a potential small advantage of text. While we expected a stronger corrective effect for images based on their ability to capture attention (Bakhshi et al., 2014; Keib et al., 2018), images can also lack conceptual nuance that is easily conveyed by language. Specifically, facial expressions can be ambiguous given that interpretation depends on characteristics of individuals and context (Barrett et al., 2011; Besel & Yuille, 2010; Fang & Li, 2024), and such ambiguities may limit how clearly (dis)endorsement is conveyed. Another possible explanation, though speculative, concerns the perceived credibility of the source. Text-based skeptical comments may be seen as more deliberate and reasoned, whereas images may be associated with humor, memes, or informal online interactions, such that the comment source may be perceived as more or less credible depending on what format they chose, with flow-on effects on persuasiveness (Briñol & Petty, 2009; Pornpitakpan, 2004).

The finding that negational comments produced the strongest belief reduction demonstrates that direct rebuttals that convey some level of certainty and indicate the existence of factual counterevidence (even though not providing any) are more powerful than expressions of doubt or uncertainty. This is in line with research showing that direct corrections reliably reduce belief in false claims (Bode et al., 2020; Bode & Vraga, 2018; Mason & Rapp, 2026; for a review, see Ecker et al., 2022), but extends this to the case where a negational comment is not accompanied by any actual evidence. Despite the absence of evidence, negational comments may have been particularly effective because they provide an unambiguous signal that obviates the need for additional deliberation that skeptical comments might require.

The fact that supportive cues were generally associated with greater perceived claim accuracy aligns with previous research demonstrating that consensus signals can enhance truth perceptions (e.g., Alister et al., 2025; Butler et al., 2023; Lewandowsky et al., 2019). Our results extend this work by demonstrating the efficacy of even minimalist cues to convey broader social endorsement. It should be noted, however, that exploratory post hoc contrasts suggested that supportive comments increased belief primarily in conditions where dissent was also present. While this should be interpreted with caution, a tentative explanation is that supportive cues may be more salient in the presence of a dissenting comment. Supportive comments may therefore serve as a buffer against the belief-reducing impact of dissent, rather than boosting claim belief per se. In other words, minimalist supportive comments may have little impact on claim perceptions unless people are motivated to seek an additional signal in the presence of dissent.

In terms of practical implications, the present findings show that user comments—whether supportive or dissenting—provide input into veracity evaluations, thereby influencing belief in online claims. As platforms move away from professional fact-checking (Augenstein et al., 2025; Borenstein et al., 2025), understanding how user comments shape beliefs is increasingly important. Existing research has established the potential utility of wisdom-of-the-crowd-based fact-checking (Allen et al., 2021) and community annotations (Drolsbach et al., 2024; Slaughter et al., 2025); however, relying on community consensus for fact-checking can be problematic, as polarized debates hinder agreement and leave the process open to strategic manipulation (Solovev & Pröllochs, 2025; Truong et al., 2025; Wirtschafter & Majumder, 2023; Yao et al., 2024). Against this backdrop, our findings point toward the risk that expressions of skepticism (as well as non-evidence-supported negations) may be directed at true claims, reducing their perceived veracity and undermining trust in reliable information. Thus, unmoderated reliance on user opinion could amplify misleading signals rather than correct them—an argument for platform policies or even regulatory mechanisms to ensure user-generated corrections remain supplemented by stronger approaches such as professional fact-checking.

By and large, platforms have avoided negative signaling mechanisms such as “dislike” buttons; in part, this may be due to the ambiguity inherent in such signals, which can indicate genuine disliking of undesirable message content but also disbelief or disapproval of a message being shared (Fay et al., 2025). Providing social-media users with more nuanced feedback and evaluation options may be a useful avenue for reducing signal ambiguity—for example, a platform could implement a “disbelief” button alongside a “dislike” button. Educating users about the effectiveness of such strategies may empower them to apply skeptical judgment when encountering dubious claims online, potentially fostering a culture of critical engagement in which users are encouraged to actively evaluate content credibility (Badrinathan & Chauchard, 2024; Bode & Vraga, 2018). One advantage of visual signals such as emojis or facial expressions in this context is that they can transcend language barriers, enabling application in diverse global contexts.

The present findings should be interpreted in light of some limitations. First, this study focused on dubious yet plausible claims, so it is unclear whether the findings generalize to claims outside the moderate-credibility range or claims about politically sensitive topics. Future research should therefore examine claims across a broader credibility spectrum and include potentially polarized content. Second, participants in this study rated their belief in multiple claims successively; while this mirrors how social-media users continually evaluate the accuracy of numerous claims to decide if and how to react, making multiple explicit judgments may have prompted participants to engage in more analytic processing than they usually would, potentially increasing their sensitivity to social (dis)endorsement. Third, dissenting comments were always presented last to avoid ambiguity, which may have introduced order effects. Future research could explore alternative formats that avoid both ambiguity and strict ordering. Fourth, only dissent was expressed visually and the ratios of support versus dissent were tightly constrained, whereas real social-media environments will feature both supportive and dissenting images in various proportions, which could be explored in future work. Finally, participants rated their belief in dubious claims while still viewing the accompanying comments; although this procedure reflects how social-media users evaluate content at the point of exposure, it is unclear whether the effect of comments persists over time or is, in part, due to demand characteristics. Future research should thus examine whether the effects of comments persist by testing claim belief after a delay.

## Conclusion

To conclude, this study demonstrated that user comments significantly shape belief in dubious claims. Even minimal expressions of textual or image-based skepticism were sufficient to lower claim belief, with brief negations that lacked evidentiary support even more effective, demonstrating that dissent can operate without evidence or elaboration. While user comments have potential as scalable accuracy signals, they also pose risks of diminishing trust in reliable information.

## Supplementary Information

Additional file 1.

## Data Availability

The data and materials for this study are available at: https://osf.io/5nbcu/. This research was not preregistered.

## Footnotes

1 We use misinformation here as an umbrella term encompassing all types of inaccurate or misleading information (Ecker et al., 2022). We note that misinformation has been linked to various adverse outcomes, including the formation of misconceptions, risky health behaviors, political polarization, and public distrust in institutions (Ecker et al., 2025; Schmid et al., 2023; Whitten-Woodring et al., 2020).

2 Some previous studies did not clearly separate these forms of dissent—for example, Kluck et al. (2019) used both negational and skeptical statements in the same condition. Conceptual separation is important to better understand whether different forms of dissent have distinct impacts on beliefs.

3 It should be noted that the main analyses were conducted using mixed-effects models; however, the ANOVA framework is considered suitable for approximating power to detect fixed effects in balanced designs (see Murayama et al., 2022). The estimated effect size was based on Butler et al. (2023), which found significant effects of social endorsement, such that misleading social-media posts were believed more and fact-checks produced greater belief updating when they were presented with a high (vs. low) number of likes.

4 A mixed-effects analysis with claim accuracy (true, false) as an additional predictor revealed that the reported effects were observed for both true and false claims. Accuracy significantly interacted with social endorsement (*p* =.023, *d* = 0.05), indicating that although supportive comments generally increased belief, this effect was larger for true claims (*F*[1, 93339] = 24.22, *p* <.001, *d* = 0.17) than false claims (*F*[1, 93339] = 9.12, *p* =.003, *d* = 0.08). Claim accuracy did not significantly interact with dissent (*p* =.617, *d* = 0.03), nor was there a significant three-way interaction (*p* =.579, *d* = 0.03). The full model results are reported in the Supplemental Materials.

5 Initial modeling additionally included a random effect for image; this had no significant effect and did not improve model fit and was thus dropped for the sake of parsimony and interpretability.
