# Supplementary material for: You sure about that? The effects of textual and image-based Skepticism on belief in dubious social-media claims
Source: Cogn Res Princ Implic. 2026 Mar 30;11:30. doi: 10.1186/s41235-026-00725-x (PMC13035968; doi:10.1186/s41235-026-00725-x)
Supplement: Supplementary file 1 — Additional file 1. [file 41235_2026_725_MOESM1_ESM.docx]

**Supplemental Materials**

**Table S1.1**

*Mixed-Effects Model Results with Accuracy*

| Term | *df* | *F* | *p* | *d* |
| --- | --- | --- | --- | --- |
| Social Endorsement | (1, 9339) | 33.20 | .000 | 0.12 |
| Dissent | (3, 9339) | 17.26 | .000 | 0.15 |
| Accuracy | (1, 46) | 9.31 | .004 | 0.90 |
| Social Endorsement × Dissent | (3, 9339) | 1.78 | .148 | 0.05 |
| Social Endorsement × Accuracy | (1, 9339) | 5.17 | .023 | 0.05 |
| Dissent × Accuracy | (3, 9339) | 0.60 | .617 | 0.03 |
| Social Endorsement × Dissent × Accuracy | (3, 9339) | 0.66 | .579 | 0.03 |
